# Advancing Meibography Assessment and Automated Meibomian Gland Detection Using Gray Value Profiles

**DOI:** 10.3390/diagnostics15101199

**Published:** 2025-05-09

**Authors:** Riccardo Forni, Ida Maruotto, Anna Zanuccoli, Riccardo Nicoletti, Luca Trimigno, Matteo Corbellino, Sònia Travé-Huarte, Giuseppe Giannaccare, Paolo Gargiulo

**Affiliations:** 1 Institute of Biomedical and Neural Engineering, Reykjavik University, 102 Reykjavik, Iceland; riccardo21@ru.is (R.F.); ida23@ru.is (I.M.); paolo@ru.is (P.G.); 2 Espansione Group, 40050 Bologna, Italy; anna.zanuccoli@espansione.it (A.Z.); nicoletti@espansione.it (R.N.); luca.trimigno@espansione.it (L.T.); matteo.corbellino@espansione.it (M.C.); 3 Optometry and Vision Sciences Research Group, Aston University, Birmingham B4 7ET, UK; s.trave-huarte@aston.ac.uk; 4 Eye Clinic, Department of Surgical Sciences, University of Cagliari, 09123 Cagliari, Italy; 5 Department of Science, Landspitali University Hospital, 105 Reykjavik, Iceland

**Keywords:** gray value analysis, meibography, meibomian glands, dry eye, meibomian gland dysfunction

## Abstract

**Objective**: This study introduces a novel method for the automated detection and quantification of meibomian gland morphology using gray value distribution profiles. The approach addresses limitations in traditional manual and deep learning-based meibography analysis, which are often time-consuming and prone to variability. **Methods**: This study enrolled 100 volunteers (mean age 40 ± 16 years, range 18–85) who suffered from dry eye and responded to the Ocular Surface Disease Index questionnaire for scoring ocular discomfort symptoms and infrared meibography for capturing imaging of meibomian glands. By leveraging pixel brightness variations, the algorithm provides real-time detection and classification of long, medium, and short meibomian glands, offering a quantitative assessment of gland atrophy. **Results**: A novel parameter, namely “atrophy index”, a quantitative measure of gland degeneration, is introduced. Atrophy index is the first instrumental measurement to assess single- and multiple-gland morphology. **Conclusions**: This tool provides a robust, scalable metric for integrating quantitative meibography into clinical practice, making it suitable for real-time screening and advancing the management of dry eyes owing to meibomian gland dysfunction.

## 1. Introduction

Dry eye disease (DED) is a complex and multifactorial condition that is frequently driven by meibomian gland dysfunction (MGD), which causes instability of the tear film. The meibomian glands play a critical role by secreting the lipid layer of the tear film, preventing excessive evaporation, and maintaining a healthy and lubricated ocular surface [1,2]. Accurate assessments of these glands are pivotal for the effective diagnosis and treatment of MGD, and infrared meibography has emerged as one of the most widely used imaging techniques for this examination.

Meibography is a non-invasive imaging technique that visualizes the structure of the meibomian glands using infrared light. This allows clinicians to detect abnormalities such as: gland shortening, dropout, blockage, and tortuosity by capturing detailed images of the glands inside both eyelids [3]. However, traditional meibography analysis is often performed manually, leading to time-consuming, subjective interpretations that suffer from high interobserver variability [4]. Addressing these limitations through automation is essential for improving diagnostic accuracy and consistency.

To overcome the challenges of manual meibography evaluation, semi-automated and automated techniques have been developed. These methods often employ image segmentation and feature extraction algorithms to differentiate meibomian glands from surrounding tissues [5]. More advanced solutions involve machine learning, particularly deep learning models like convolutional neural networks (CNNs), which have demonstrated high accuracy in detecting gland abnormalities [6]. While deep learning techniques have achieved impressive accuracy in detecting meibomian glands, significant practical limitations remain, such as the extended computational time required for both training and inference, which hinders real-time application. For instance, CNNs, though effective for segmenting glands and detecting gland dropout, require substantial computational resources, making them unsuitable for use in fast-paced clinical settings where immediate feedback is critical [7]. These delays are exacerbated by the need for pre- and post-processing steps, further complicating their integration into clinical workflows.

To address these limitations, this study proposes a novel method for the quantitative evaluation of meibography images that reduces computational time while maintaining high accuracy. Our approach leverages gray value distribution profiles to detect and characterize meibomian glands automatically, providing a feasible solution for real-time analysis and integration into clinical workflows.

Pixel brightness, or gray value, reflects a tissue’s response to light and varies based on the imaging technology used, such as X-ray absorption in computed tomography scans or magnetic properties in magnetic resonance imaging [8,9]. Recent advances in radiomics, which examine pixel intensity and spatial relationships, have shifted focus from broad imaging to key regions of interest, enabling more precise, evidence-based analyses of pathological tissues [10,11]. For instance, radiodensity measures in skeletal muscle have been linked to predicting sarcopenic degeneration and cardiovascular diseases [12,13,14]. In meibography, infrared imaging captures the thermal responses of meibomian glands, requiring precise differentiation between glands and the surrounding tarsus. However, due to tissue overlap and light exposure, this distinction can be challenging. To overcome this limitation, a local approach is proposed. To recognize glands, the alternation between brighter spots (actual glands) and darker one (tarsus, background) is exploited: following a curved line from the left to the right side of the eyelid, a gray value swing may be observed, where the peaks match with glands and the valleys match with the background.

The purpose of this study is to develop an automated instrumental measurement for detecting and quantifying single- and multiple-meibomian-gland morphology in the lower eyelid.

## 2. Materials and Methods

### 2.1. Study Setup and Data Acquisition

The study enrolled 100 volunteers (mean age 40 ± 16 years, range 18–85, 53% female) without a definitive diagnosis of DED but reporting a variable degree of ocular discomfort symptoms. Exclusion criteria were related to a history of epilepsy, and metal implants in the facial area. Subjects were visited at 3 time points (day 0, day 14, and day 28) for the evaluation of (i) the Ocular Surface Disease Index (OSDI) questionnaire for the assessment of ocular discomfort symptoms [15] and (ii) infrared images of the meibomian glands (Me-check^®^, Espansione Group, Bologna, Italy).

The cohort was split into 10-year age groups, and a description is reported in Table 1.

After the baseline evaluation, patients were treated once with Low-Level Light Therapy (LLLT) technology using the My-mask^®^ device (Espansione Group, Bologna, Italy) for 15 min with their eyes closed [16]. An infrared picture of meibomian glands was taken again after the LLLT session.

The OSDI score was categorized in 4 classes, as follows: normal (0–12), mild (13–22), moderate (23–32), and severe (33–100) dry eye [17]. The meibography of the lower eyelid was taken in a dark environment to minimize the effect of external light and reflection on image quality. The Supplementary Materials (S1) report the analysis of the LLLT effect on ocular discomfort symptoms and meibomian glands.

### 2.2. Meibomian Gland Profiling

**Figure 1:**
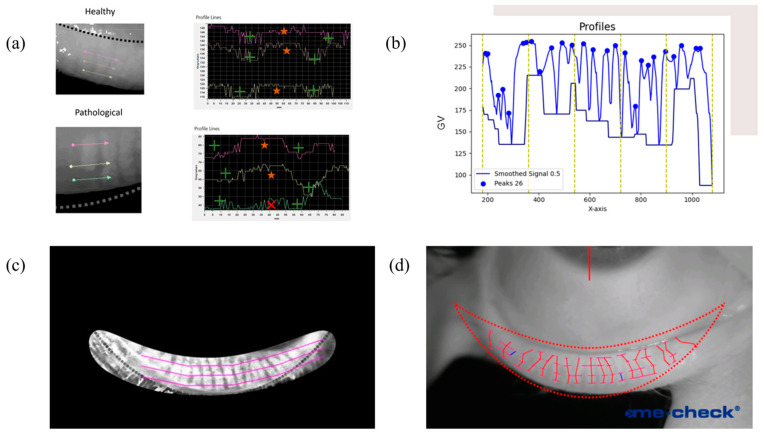
Major steps for gland detection: (**a**) local image of how gray value gradient can be associated with the presence of a gland (star) or a missing gland in a pathological condition (X) and the background (+); (**b**) the gray value profile extracted from a single line, filtered and with the peaks recognized; (**c**) how the sampling was carried out to determine the presence and size of meibomian glands; (**d**) the result, highlighting the skeleton of the glands and the width in corresponding nodes.

The proposed approach relies on the brightness gradient obtained while crossing the meibomian glands from one side to the other at different heights, knowing that a pathological eyelid may show shortening in some glands. Figure 1a reports an example of how the signal shape is when extracted from a healthy and pathological region of an everted eyelid. After a moving average filter with a window size equal to 200 steps, a chart of gray values over the x position was drawn, and it represented the alternation of glands and tarsus below the segments as the gradient of the gray value (Figure 1b). The number of glands detected by the segments was estimated by the number of peaks found on the signals. Since the number of peaks varied over the lines, to calculate the actual number of glands and their length, Dijkstra’s algorithm was introduced. Dijkstra’s algorithm is a weighted-graph algorithm that finds the shortest path from a given node source to every other node [18]. In a weighted graph, the shortest path is the collection of edges with the minimum total weighted sum; in our case, the graph is built in a way that every path represents a gland. The eyelid was isolated with a pre-constructed binary mask that replicated the Me-check^®^ built-in alignment, and 4 lines were used to extract gray values at different heights (Figure 1c). Each line was mapped into a layer of the graph, and each peak was mapped into a node. Nodes from the adjacent layer were fully connected with arcs weighted by the distances between the two nodes. To reconstruct the elongated shape of a gland, the minimum path was found (Figure 1d). First, all the layers were used to find the full-length glands. In the subsequent iteration, the upper layer was removed as well as nodes already associated with a gland, and the logic was reapplied to detect shortened glands. Each gland was then labeled as long (L), medium (M), or short (S) based on the number of layers involved, and therefore, the length became discretized. A counting step was introduced for each group as the sum of the number of glands belonging to that discretized length (N_L_, N_M_, and N_S_).

The width of the peak provided an estimation of the width of the glands at 50% of the peak height. In addition, the average width of glands per class x (L, M, or S) was computed as follows:

$${AveWidth}_{x}=\frac{\sum_{1}^{n}{Width}_{x,i}}{N_{x}}$$

where i from 1 to *n* means that the sum was performed on all the glands of that group.

### 2.3. Profiling Automation

**Figure 2:**
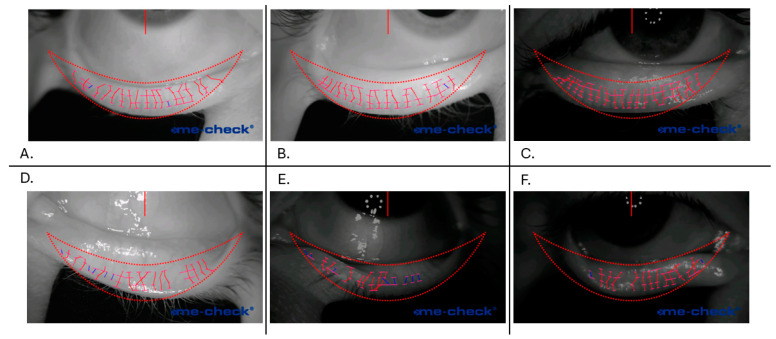
The algorithm’s output for different types of images. The first row (**A**–**C**) reported well-segmented glands, in two opposite illumination conditions. The second row (**D**–**F**) reported the more common mistakes where glands were misrecognized due to reflection artifacts (**E**,**F**) or incorrect eyelid eversion (**D**,**F**).

An algorithm was designed to automate the profile extraction. It is based on the profile extraction previously described, and it receives input from the meibography and a mask that represents the eyelid shape; both images have a resolution of 1280 × 720 pixels. The first step is to obtain the “profile lines”, the lines underneath which the gray value is extracted. To achieve this, the mask is divided into 5 different vertical zones, and the size of the foreground is divided into percentages (0.10, 0.45, 0.55, and 0.75) downwards. Each point is connected to one of the subsequent zones and at the same height. In this way, we obtained piecewise continuous lines that accommodated the eyelid shape (Figure 2).

The input image undergoes a filtering process to enhance the contrast. First, the dark images are corrected by moving the histogram in a way that it is centered around the value 100 (considering the gray value in a range from 0 to 255). Then, several filters are applied to enhance the contrast between the glands and the foreground. The first filter is a Canny with an aperture size of 3, a lower threshold of 0, and a higher threshold of 255, the two extremes. It was found that this enhances the light reflex on the eyelid, so the effect can be mitigated by extracting the burned pixels (white) of the obtained images and substituting those pixels with a value in the original image. The value is evaluated since the reflection is related to a localized and more humid zone of the eyelid. Therefore, these areas can be considered as connected components, filling the inside with a mean value evaluated from the contour pixels. Next, the dark pixels are transformed to pure black with a threshold of 50, as the intensity is enhanced in terms of the contrast between light pixels and darker ones. The image histogram is then equalized through the contrast-limited adaptive histogram equalization (CLAHE) technique with a clip limit of 12 and a 20 × 20 grid, and the goal is to remove the illumination gradient that can affect the algorithm’s performance. Then, a gray value closing morphological transformation is applied to remove small dark holes in the bright foreground of glands. The structuring element is a circle with a diameter of 5 pixels. Finally, the histogram is equalized to stretch the values among the full range of representation and separate the background from the foreground.

From the filtered image, the gray values are extracted from the profiles, the curves are smoothed with a linear Savitzky–Golay filter with a window length of 11, and the peaks with prominence of 0.1 and a minimum width of 2 are considered valid.

To reduce the number of double detections in the same gland, peaks closer than 10 pixels cannot be a gland, so they are fused into a new peak localized in the middle with a width that is the sum of the two older ones. Sometimes, peaks that do not represent a gland can be recognized because of their high width, so we removed them with a threshold of 90 pixels.

The algorithm’s core is a graph that allows the peaks from different profiles to be connected to trace the glands. Each peak can be represented as a node, and a group of them is a layer; the nodes contained in a layer come from the same profile and are not connected at all. Since the profiles are ordered, the layers are also “ordered” in the sense that each node of the first profile (the upper or the lower, depending on how you build the graph) is connected to every node of the second profile and only to those nodes with direct edges. The nodes in the second layer are connected to the nodes in the third one, and so on. It is worth noticing that each node in the second layer has inward edges from every first layer’s node. The edges are weighted with the Euclidean distance between the two nodes that they connect. Two special nodes are added to this graph, and they are the “supersource” and the “supersink”; the first one is connected to every node of the first layer with an edge weight of 0, and the second one is connected to every node of the third layer with an edge weight of 0.

The two special nodes serve as the Dijkstra algorithm’s starting and ending points, searching for the shortest path from the source to the sink. Considering the meaning of the edges’ weights, the shortest path is the connection between nodes of different layers that are generally “closer”: the gland and its length is the sum of the edges’ weights, which is the path length itself. The selected nodes are removed from the graph, and the algorithm is repeated until the length of the gland is higher than a threshold fixed at 100. At this point, because the glands can shorten, the layer containing the peaks from the upper profile is removed, and either the supersink or the supersource is left without a connection. They are connected again to the new upper layer available, and the algorithm is repeated until there are at least 3 layers in the graph. In this situation, the third layer is removed, and the edges shorter than a threshold fixed to 60 pixels are considered a gland. The glands composed of only two nodes are the shortest detectable by the algorithm, but this distance can be fixed by tuning the number of profiles.

In this way, we automatically obtain the number of glands, their length, and a variable number of widths linked to every gland.

### 2.4. Atrophy Index

To encapsulate the extracted information in one quantitative value, we proposed an index defined as follows:

$$Atrophy\;Index=\frac{N_{L}-(N_{M}+N_{S})}{N_{T}}$$

where N_L_ is the total number of long glands from that image, N_M_ is the number of medium length, and NS is the number of short glands. N_T_ is the sum of N_L_, N_M_, and N_S_. The atrophy index spans from −1 to 1, where the minimum is associated with a subject having all the glands shortened, and the maximum is a subject where all the glands are healthy and cover the entire eyelid in length.

### 2.5. Statistical Analysis

The atrophy index and AveWidth parameters were computed for each eye for each subject. A Pearson correlation analysis was performed with OSDI scores to search for possible relations between self-reported statuses and objective measurements. In addition, a Kruskal–Wallis test, due to the non-normality of the data, was performed on the counted number of glands (NL, NM, and NS) over the OSDI score to verify if the number of glands was significant for the pathology.

Due to a lack of gold standards and instrumental assessment for meibography, the glands extracted by our algorithm underwent a validation process through visual inspection performed by a team of experts, including optometrists, doctors, and engineers.

## 3. Results

### 3.1. Algorithm Performance and Robustness

The number of glands is a patient-specific parameter. An example of algorithm output can be found in Figure 2, as well as in the Supplementary Materials (S2). Figure 2A–C show the algorithm’s ability to recognize glands in different bright environments, and the algorithm was able to identify most of the glands and their width. However, despite the algorithm having a built-in correction strategy to compensate for reflection spots, when this artifact was too heavy, the computation failed by missing glands and/or their length (Figure 2D,F). In addition, the output strongly depends on the quality of the eyelid eversion, such as prolapsed conjunctivae (Figure 2D) and incomplete eversion (Figure 2E,F), leading to missing glands and/or due to practitioner eversion expertise.

**Figure 3:**
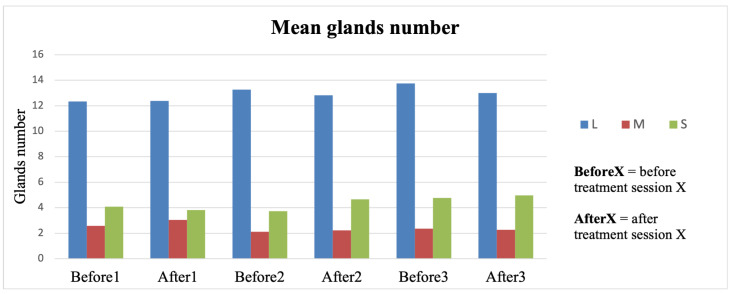
Mean number of meibomian glands in the lower eyelid during the different time points over the entire population.

The same number of glands among the appointments should be counted to validate the algorithm; indeed, in Figure 3, the number of mean glands recognized in each visit shows almost no variation within the same gland group.

This number is extracted by keeping the same algorithm parameters in the analysis of each image because this number is highly dependent on them. Indeed, the number of profiles and their location, with respect to the eyelid and gland positioning, is a crucial factor in correctly recognizing the glands.

### 3.2. Atrophy Index and OSDI Score

The outliers were handled by using only good-quality images and manually inspecting the algorithm’s output. However, extreme values outside the 5th and 95th percentiles were excluded from the subsequent analysis.

The number of glands from each grade of severity of the disease was reported as not statistically different (Kruskal–Wallis *p*-value > 0.05 for N_L_, N_M_, and N_S_ over the grades normal, mild, moderate, and severe).

**Figure 4:**
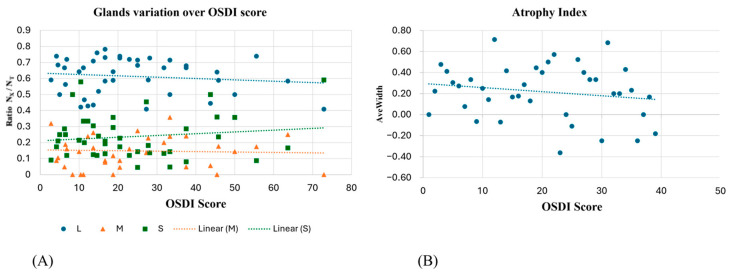
(**A**) The ratio of the number of long/medium/short glands over the total number per subject and their trends; (**B**) the atrophy index correlation with OSDI score.

A correlation analysis of the normalized number of glands in each class on the OSDI score reported Pearson coefficients of −0.13, −0.04, and 0.14, respectively, for long, medium, and short glands (Figure 4A). When combined into the atrophy index, the correlation analysis on the OSDI obtained from the measured cohort reported a Pearson coefficient of −0.28 (*p*-value = 0.07), and the correlation can be seen in Figure 4B.

**Figure 5:**
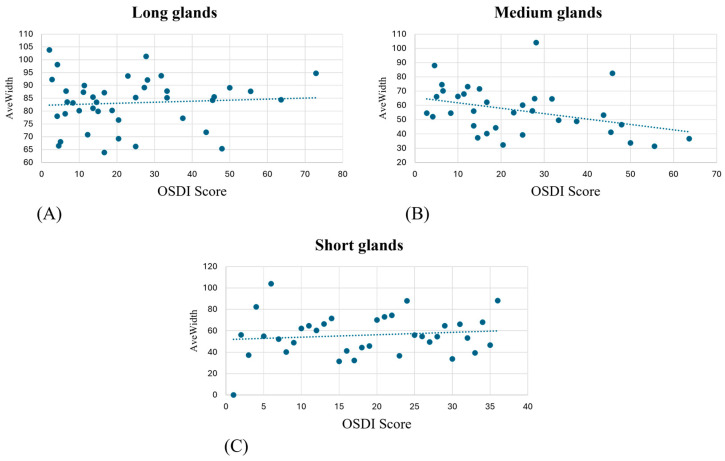
AveWidth variation in OSDI score at first visit for detected long (**A**), medium (**B**), and short (**C**) meibomian glands.

AveWidth showed no correlation with OSDI score when extracted from long glands (Figure 5A) but showed a progressive decrease for the medium gland (Figure 5B) and an increase for short glands (Figure 5C).

## 4. Discussion

This study describes a novel method for the analysis of meibography based on quantitative descriptors from the gray value distribution and develops the atrophy index that, to the best of our knowledge, represents the first instrument for the evaluation of meibomian gland morphology. The proposed methodology, based on gray value profiles, successfully identified and quantified glands, offering an objective and automated approach compared to traditional methods that rely on visual evaluation. This innovation addresses a crucial need in the field, where quantitative assessment tools are limited, and most of the current analysis remains qualitative and subjective.

Our algorithm successfully differentiated between gland morphology by detecting distinct gray value peaks corresponding to meibomian glands. This segmentation approach enabled a more detailed evaluation that is particularly valuable in assessing the severity of gland shortening/loss or atrophy, which could be associated with several clinical conditions.

The analysis conducted between the gland’s widths/atrophy index and OSDI score failed to find a correlation. This result may be explained by the subjective nature of the OSDI questionnaire and its short-term variability, even after a single LLLT session. Conversely, as reported in Figure 2, the atrophy index does not change over 2 months of follow-up, independently of the efficacy of the LLLT, requiring longer time intervals for frank longitudinal changes.

Recent studies in deep learning-based segmentation, such as the Mask R-CNN and U-Net architectures, have shown excellent performance in gland segmentation and dropout detection. These models utilize large, annotated datasets to train their algorithms and can rapidly segment meibomian glands [19,20]. However, one of the key limitations of these approaches is the substantial computational power and time required for both training and inference, which delays results and reduces their applicability in fast-paced clinical environments [7]. Additionally, these models often require significant pre- and post-processing steps to refine gland segmentation, further complicating their integration into commercial devices for everyday clinical practice [21,22].

In contrast, our proposed gray value distribution-based method reduces the computational complexity, providing a robust evaluation of single- and multiple-gland morphology. This allows for real-time analysis, providing immediate feedback to clinicians, which is critical for timely decision making at diagnosis and throughout therapy during patient evaluations. Moreover, our approach does not require large training datasets, making it more accessible for clinical settings where data collection and annotation are challenging.

The key innovation of our algorithm is its ability to quantify morphological metrics such as gland length, width, and atrophy index, which are crucial for objectively tracking the progression of MGD. Our method allows a direct calculation of gland morphology from the gray value profiles.

In terms of clinical impact, the real-time capabilities of our method provide significant advantages. Unlike deep learning models, which require dedicated hardware and extensive computational resources [18], our algorithm can be implemented on standard clinical equipment without sacrificing performance. This facilitates its integration into existing clinical workflows and commercial meibography devices, ultimately improving patient care by delivering rapid and reliable results. Additionally, the automated nature of our method eliminates the subjectivity and potential for human error inherent in manual meibography analysis, increasing diagnostic accuracy and reproducibility across different clinical environments. This approach has the potential to substantially improve the diagnosis and management of MGD, making advanced meibography analysis more accessible and practical for a broad spectrum of eye care professionals.

The limitations of the proposed study concern the high variability and complexity of meibography since it might be difficult to assess the exact point where the eyelid ends. Thus, an expert operator is still essential. For these reasons, future work may include more subjects and an ad hoc binary mask to better isolate the everted eyelid.

The present work advances state-of-the-art techniques for the imaging of meibomian glands, and the main benefits and novelties are (i) a pixel-based automatic algorithm for the identification and localization of meibomian glands and (ii) a multiscale morphological parameter extraction methodology where information can be retrieved from a single gland up to the entire eye lid gland population.

## 5. Conclusions

In conclusion, this study introduces a novel, automated approach for the detection and quantification of meibomian glands using gray value distribution profiles. The algorithm developed demonstrates high performance in terms of computational efficiency and gland recognition ability, making it suitable for real-time clinical use in diagnosing and managing MGD. By providing a quantitative, objective method to assess gland morphology, this approach offers significant advantages over traditional manual and deep learning techniques, particularly in fast-paced clinical environments. This innovation represents a step forward in the integration of meibography into everyday ophthalmic practice.

The algorithm described in this study is currently patent pending, further underscoring its unique contribution to the field. As we continue to refine this technology, future research should explore its broader applications and potential to enhance patient care.

## Figures and Tables

**Table 1:** Demographic and clinical characteristics of the study population by age group. The table reports average age (years old), gender distribution (female/male), and baseline Ocular Surface Disease Index (OSDI 1) scores for each age group.

	20–30 (Years)	30–40 (Years)	40–50 (Years)	50–60 (Years)	>60 (Years)
**Age (mean)**	27.0	35.8	43.6	53.3	67.7
**Gender (F/M)**	24/16	6/5	2/5	9/6	5/7
**OSDI 1**	18.5	26.6	20.4	22.4	27.0

## Data Availability

The datasets generated and analyzed during the current study are not publicly available due to privacy concerns and regulations regarding patient data protection, but de-identified data are available from the corresponding author upon reasonable request and with appropriate institutional review board approval.

## Supplementary Materials

The following supporting information can be downloaded at https://www.mdpi.com/article/10.3390/diagnostics15101199/s1. S1 contains the methodology and results about the effect of the LLLT. S2 contains additional images as output examples of the proposed algorithm.
