# Supplementary material for: Advancing Meibography Assessment and Automated Meibomian Gland Detection Using Gray Value Profiles
Source: Diagnostics (Basel). 2025 May 9;15(10):1199. doi: 10.3390/diagnostics15101199 (PMC12110248; doi:10.3390/diagnostics15101199)
Supplement: Supplementary file 1 [file diagnostics-15-01199-s001.zip › diagnostics-3532854-supplementary.pdf]

S1 – Low-Level Light Treatment Assessment

Descriptive Statistics

A total of 100 participants provided longitudinal score data across three time points (Score1, Score2, and Score3). The sample included multiple age groups, coded from 1 to 5, and a binary gender variable (0 = Female, 1 = Male). A linear mixed effects model was used to estimate the magnitude of each factor (Time, Gender, and AgeGroup), their interaction, and also taking into account repeated measures. The model converged successfully and explained substantial variability in the data, with a group-level variance of 20.12. The residual variance (scale) was estimated at 197.29.

Score ~ Time * Gender * AgeGroup + (1 | Subject)

Longitudinal Effects of Time

To assess changes in scores over time, a linear mixed effects model (LMM) was fitted with scores as the dependent variable. Fixed effects included Time, Gender, and AgeGroup, along with their interactions. Random intercepts were estimated for each subject to account for within-individual correlation.

The model revealed a significant main effect of Time, $\beta=-5{,}019$; $SE = 2.403$, $p = .037$, indicating that scores declined significantly over the measurement period. The decline in scores may reflect treatment natural progression and time-related effects.

Gender Differences

Gender was a significant predictor of overall scores. Compared to females (reference group), males had significantly lower overall scores, $\beta=-16{,}217$, $SE = 7.579$, $p = .032$. No significant three-way interactions involving gender were detected, although several two-way interactions approached significance.

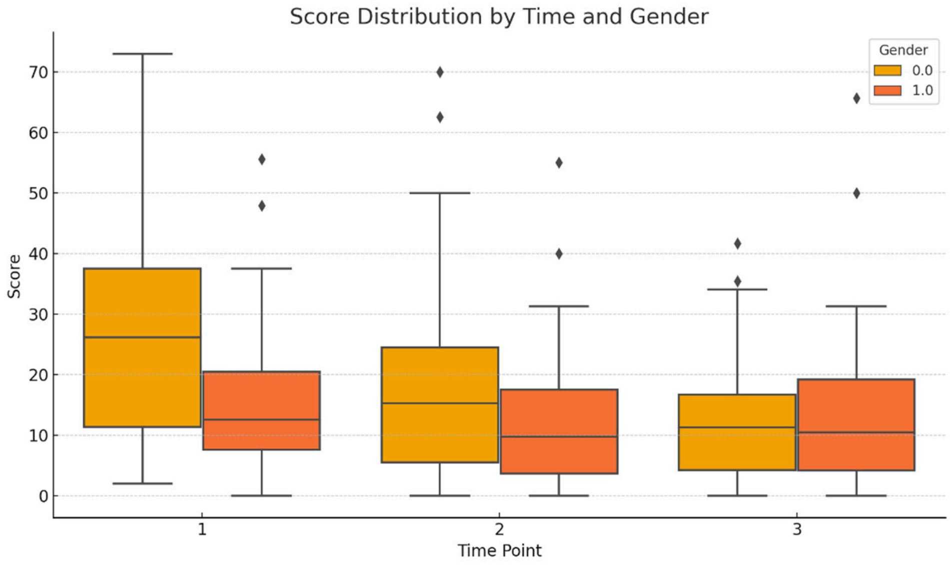

Age Group-Specific Effects

Of the five age groups, Age Group 3 demonstrated a significant interaction with Time, β=−18,621, *SE* = 9.135, p = .042. This interaction suggests that individuals in this group experienced a more pronounced decline in scores compared to others. Moreover, within Age Group 3, females showed a steeper drop over time. No other age groups showed significant interactions with Time or Gender.

S2 – Gland imaging

Correct localizations

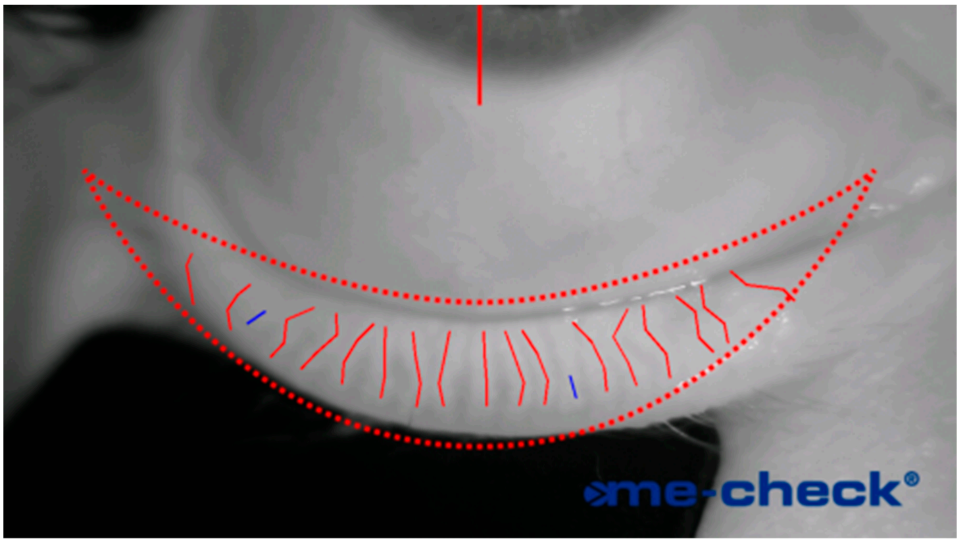

Figure S 1

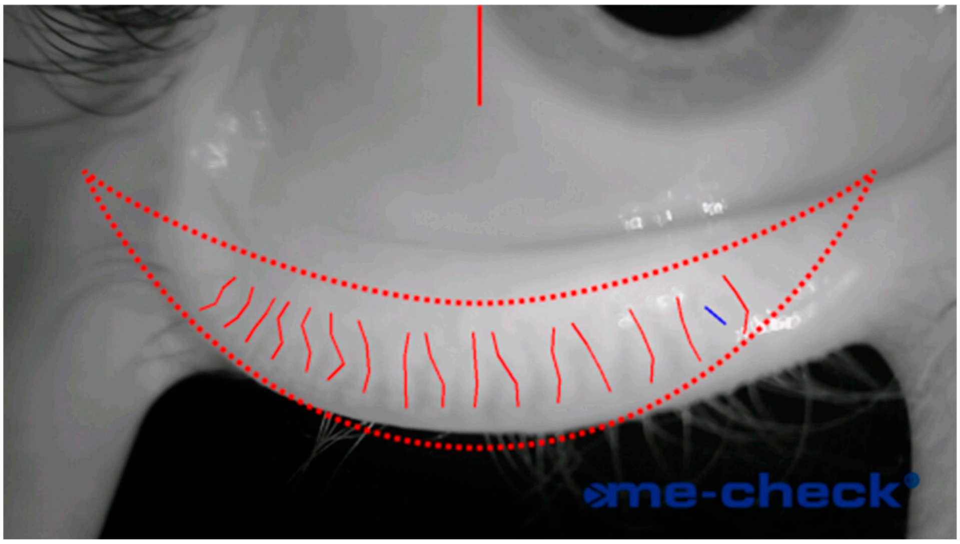

Figure S 2

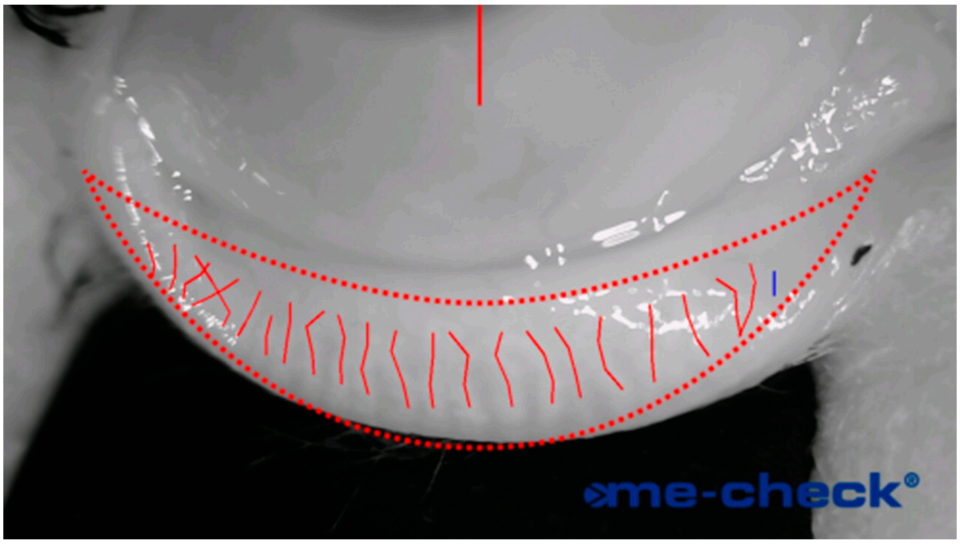

Figure S 3

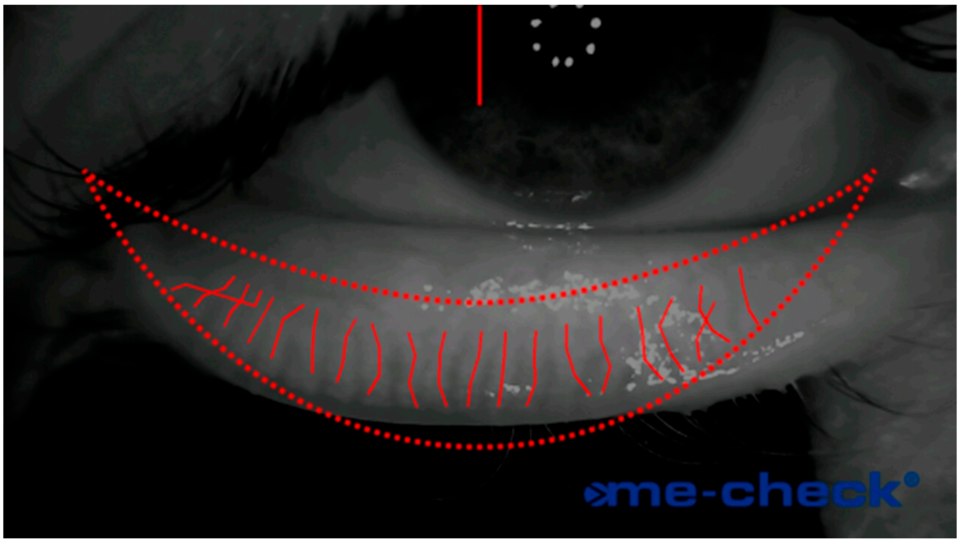

Figure S 4

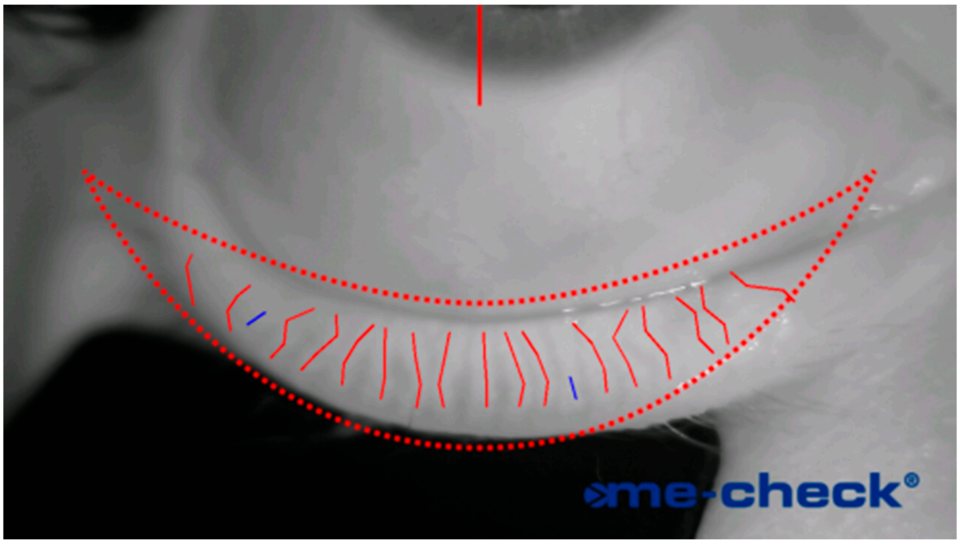

Figure S 5

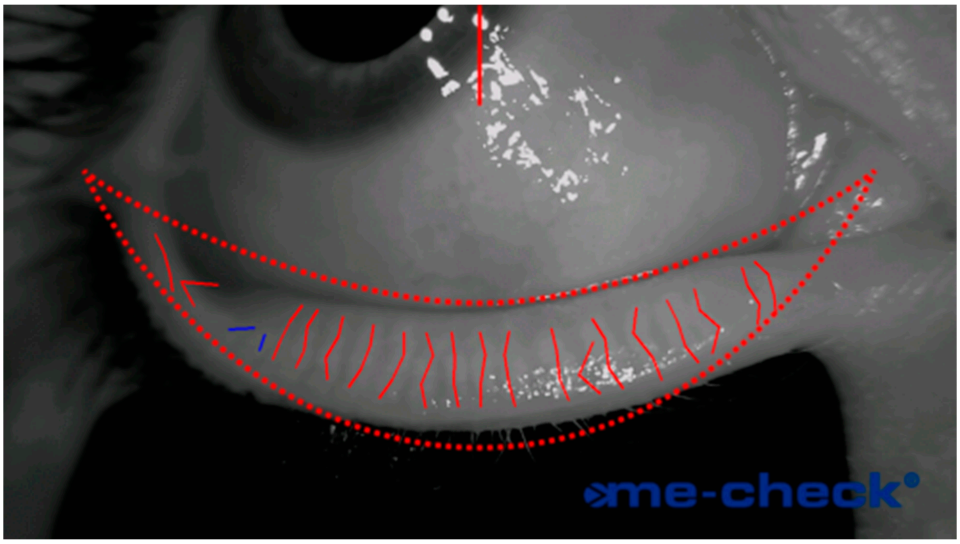

Figure S 6

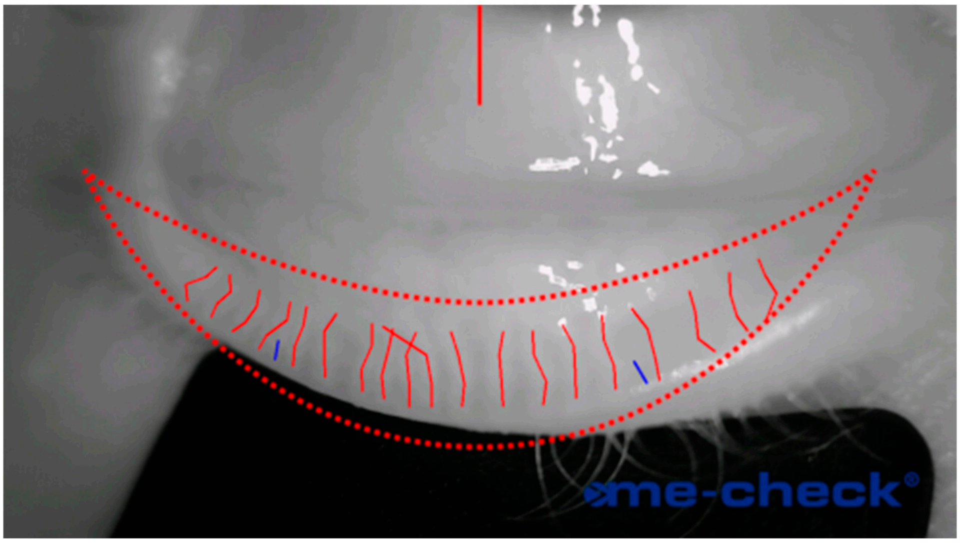

Figure S 7

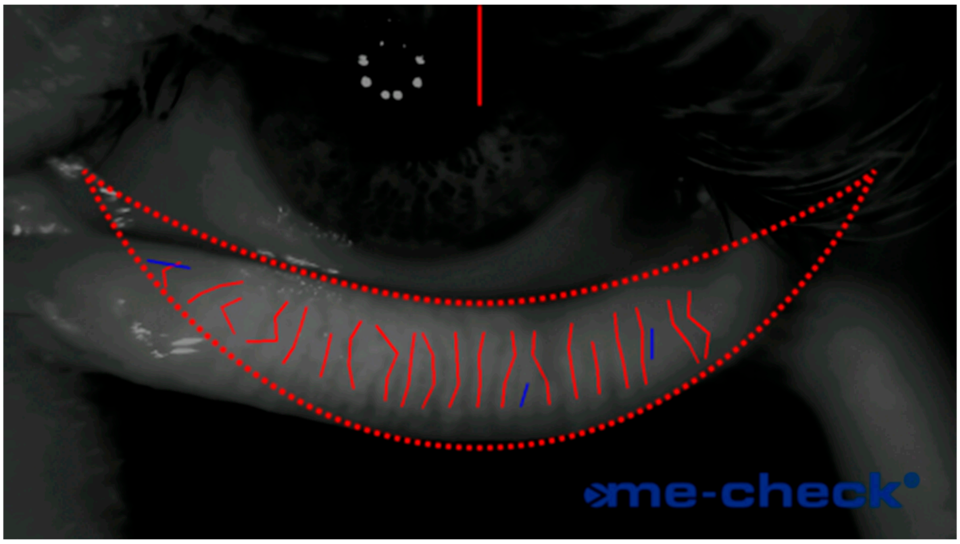

Figure S 8

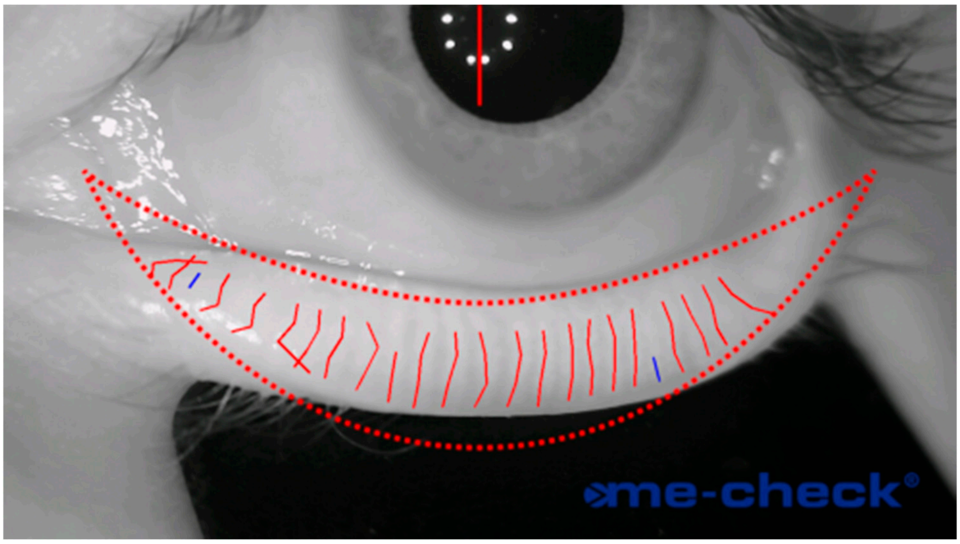

Figure S 9

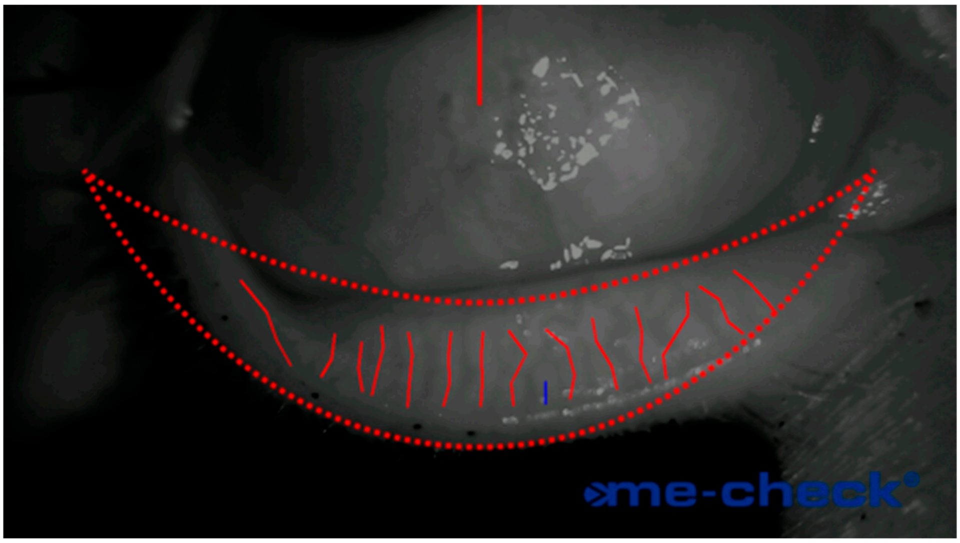

Figure S 10

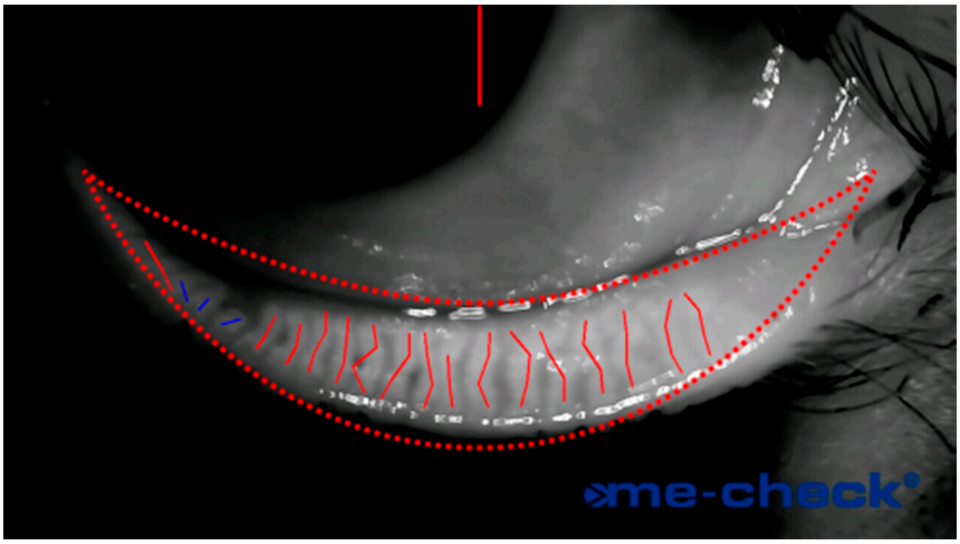

Figure S 11

Wrong localizations

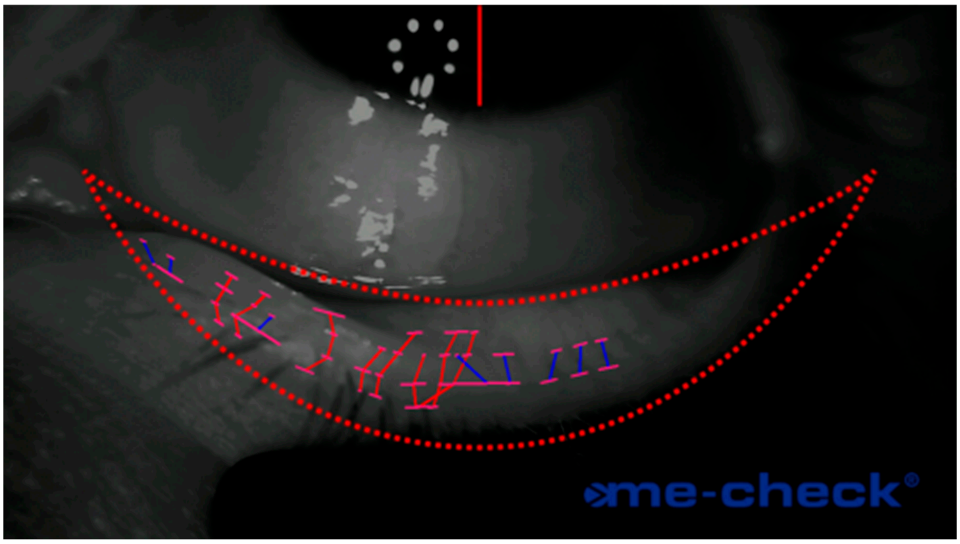

Figure S 12

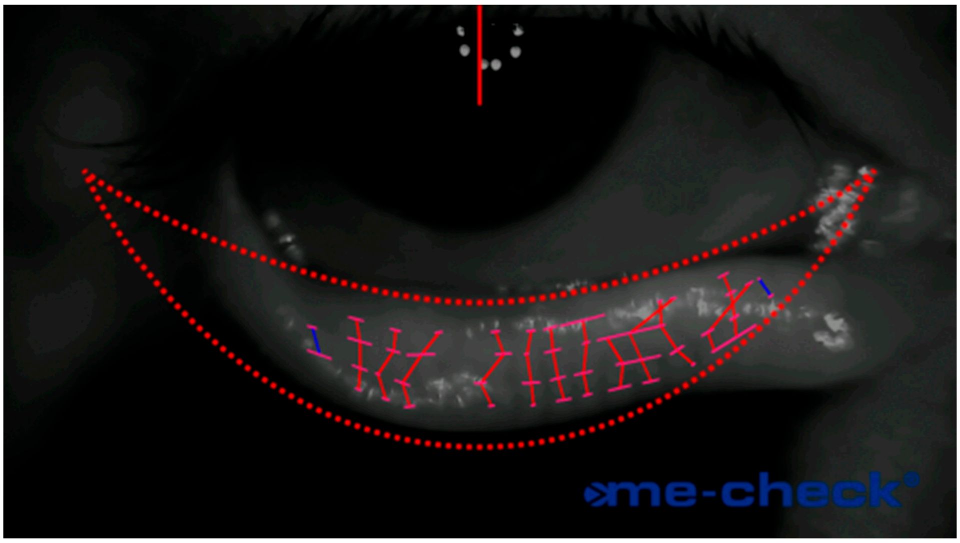

Figure S 13

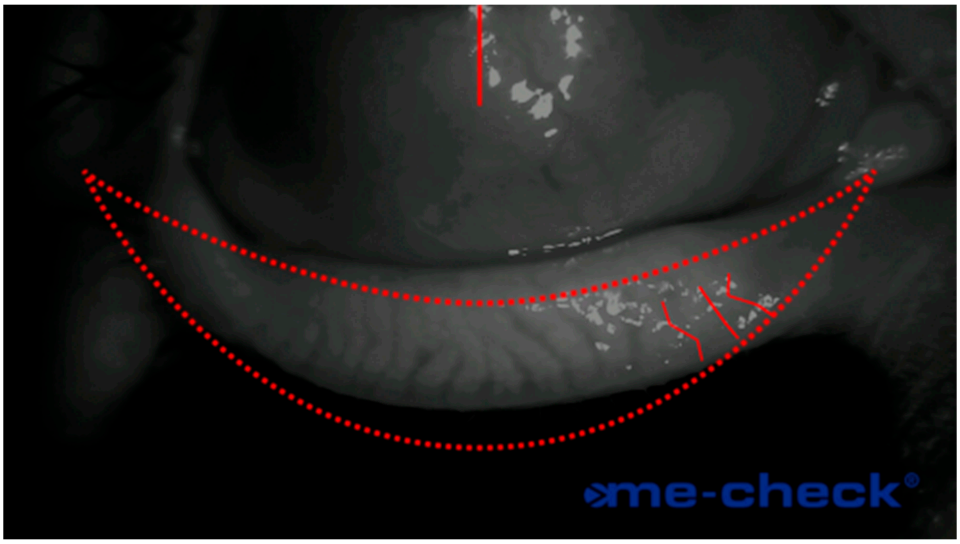

Figure S 14
